# Oxidative and O_2_ diffusive function in triceps brachii of recreational to world class swimmers

**DOI:** 10.1113/EP092299

**Published:** 2025-04-25

**Authors:** Simone Villanova, Elisa Pastorio, Andrea M. Pilotto, Alessio Marciano, Valentina Quaresima, Alessandra Adami, Harry B. Rossiter, Daniele A. Cardinale, Simone Porcelli

**Affiliations:** ^1^ Department of Movement, Human and Health Sciences University of Rome ‘Foro Italico’ Rome Italy; ^2^ Department of Molecular Medicine University of Pavia Pavia Italy; ^3^ Department of Sport, Exercise and Rehabilitation Northumbria University Newcastle Upon Tyne UK; ^4^ Department of Life, Health and Environmental Sciences University of L'Aquila L'Aquila Italy; ^5^ Department of Kinesiology University of Rhode Island Kingston Rhode Island USA; ^6^ Institute of Respiratory Medicine and Exercise Physiology, Division of Respiratory and Critical Care Physiology and Medicine The Lundquist Institute for Biomedical Innovation at Harbor–UCLA Medical Center Torrance California USA; ^7^ Åstrand Department of Physiology, Nutrition and Biomechanics The Swedish School of Sport and Health Sciences Stockholm Sweden

**Keywords:** muscle mitochondria, NIRS, sprint swimming, upper body exercise

## Abstract

This study aimed to evaluate in vivo oxidative capacity and relative resistance to O_2_ diffusion using near‐infrared spectroscopy (NIRS) in the m. triceps brachii of recreational to world class swimmers and evaluate their relationships with swimming performance. Twenty‐eight swimmers were enrolled and assigned into three subgroups according to their level: ‘recreational/trained’ (Tier 1/2; *n *= 8), ‘national’ (Tier 3; *n = *12) and ‘international/world class’ (Tier 4/5; *n =* 8). Performance was evaluated by 100 m freestyle trials. Training volume was measured by self‐reported distance (km/week). The $m{\dot{V}}_{O_{2}}$ recovery *k* of m. triceps brachii was non‐invasively estimated by NIRS through repeated intermittent occlusions under two conditions: well‐oxygenated (*k*_HIGH_) and low O_2_ availability (*k*_LOW_). The difference between *k*_HIGH_ and *k*_LOW_ (Δ*k*) was calculated as an index of relative resistance to O_2_ diffusion. FINA points and 100 m performance differed among all groups. Training volume was greater in Tier 4/5 (34.0 ± 5.5 km week^−1^) and Tier 3 (35.5 ± 11.6 km week^−1^) than in Tier 1/2 (6.4 ± 1.8 km week^−1^). *k*_HIGH_ was greater in Tier 4/5 and Tier 3 (3.18 ± 0.41 and 2.79 ± 0.40 min^−1^) versus Tier 1/2 (2.10 ± 0.36 min^−1^; all *P *< 0.002). *k*_HIGH_ correlated with FINA points, 100 m performance and training volume. ∆*k* was not different among tiers and was not associated with training volume or performance. M. triceps brachii oxidative capacity (*k*_HIGH_) was positively associated with performance and training volume in swimmers. ∆*k*, which reflects relative resistance to O_2_ diffusion, was not different among athletes. These data suggest that m. triceps brachii oxidative capacity is associated with swimming performance and that muscle O_2_ diffusing capacity exerts a similar relative resistance to O_2_ diffusive flow across swimmers.

## Abstract (highlights)

- **What is the central question of this study?**
  Do highly trained swimmers have greater muscle oxidative capacity but higher sensitivity to reductions in O_2_ availability, that is, are relatively more ‘O_2_ diffusion limited’, than recreational swimmers?
- **What is the main finding and its importance?**
  We found that m. triceps brachii oxidative capacity varied significantly among swimmers of different competitive levels, and that it was associated with both training volume and performance. However, relative resistance to O_2_ diffusion was not different among swimmers of all levels, suggesting that training‐induced adaptations to support muscle oxidative capacity were matched well to adaptations to support muscle O_2_ diffusion.

## 1 INTRODUCTION

Success in swimming competitions is a multifaceted phenomenon influenced by the interaction of several physiological, biomechanical, psychological and technical factors (Ben‐Zaken et al., 2022). The physiological demands of swimming are different for each event, but even athletes competing in sprint events (50–100 m) utilize a combination of power and endurance training and perform a greater training volume than in many other sprint sports (González‐Ravé et al., 2021). As such, competitive swimmers may be classified as endurance athletes and are characterized by high maximal oxygen uptake (${\dot{V}}_{O_{2}\max}$) (Lavoie & Montpetit, 1986). Skeletal muscles actively engaged in swimming strokes (i.e. deltoid and triceps brachii) express high oxidative enzyme activity (Gollnick et al., 1972) and abundant capillaries (Nygaard & Nielsen, 1978). However, international and world class swimmers include heavy strength training on dry land or sprint swimming drills with resistance tools in their weekly routine (Aspenes & Karlsen, 2012). Although resistance training can induce modest hypertrophy without compromising capillarization and oxidative metabolism (Green et al., 1999), it also may result in hypertrophy of type II muscle fibres, and associated reductions in capillary density, peak blood flow, mean transit time, diffusional surface area and distance, and capillary $P_{O_{2}}$, compared to what could theoretically be achieved by endurance training alone. These adaptations could lead to cause limitations in skeletal muscle O_2_ diffusion, especially at high rates of mitochondrial O_2_ demand (McCall et al., 1996). The degree to which these muscle adaptations relate to swimming performance at different competitive levels is unknown.

Skeletal muscle oxidative capacity can be assessed directly through *ex vivo* approaches such as high‐resolution respirometry (Larsen et al., 2012) or in vivo from the rate constant (*k*) of phosphocreatine recovery by magnetic resonance spectroscopy (Larson‐Meyer et al., 2000) or the ${\dot{V}}_{O_{2}}$ recovery *k* by near‐infrared spectroscopy (NIRS) (Motobe et al., 2004). Non‐invasive approaches rely on the linear relationship between isolated muscle ${\dot{V}}_{O_{2}}$ recovery *k* and the maximal rate of cellular O_2_ consumption, which is present when mitochondrial O_2_ availability is not limiting (Wüst et al., 2013). $m{\dot{V}}_{O_{2}}$ *k* from NIRS correlates with muscle oxidative capacity estimated by other methods (Pilotto et al., 2022; Ryan et al., 2014, 2012). The estimation of muscle oxidative capacity by NIRS has also been applied to evaluate muscle adaptations to exercise training. For example, $m{\dot{V}}_{O_{2}}$ *k* of the quadriceps muscle is greater in endurance‐trained athletes compared with untrained controls (Brizedine et al., 2013) and correlates with whole body ${\dot{V}}_{O_{2}\max}$ in male and female athletes (Beever et al., 2020).

We recently extended the NIRS approach to estimate muscle oxidative capacity also to allow muscle O_2_ diffusion limitation to be assessed. This was achieved by measuring the $m{\dot{V}}_{O_{2}}$ recovery *k* under limiting and non‐limiting O_2_ availability conditions. We showed that the difference between these two measurements (termed Δ*k*) is related to capillary density in biopsies of m. vastus lateralis and, therefore, allows an assessment of the relative resistance to O_2_ diffusion in vivo (Pilotto et al., 2022). A high Δ*k* value reflects a greater relative impact of O_2_ diffusion on oxidative metabolism than a low Δ*k*. When muscle mitochondrial oxidative capacity is high (as in endurance‐trained athletes), O_2_ diffusing capacity is typically also greater. However, due to the high oxidative demands, endurance‐trained muscles may become more sensitive to limitations in O_2_ diffusion, whereby relative resistance to O_2_ diffusion can become a primary limitation to maximal rates of O_2_ use (Roca et al., 1992).

Thus, this study aimed to evaluate muscle oxidative and O_2_ diffusive function in the m. triceps brachii of swimmers ranging from recreational to world class performance. We hypothesized that international and world class level swimmers would have greater muscle oxidative capacity but would be more sensitive to reductions in O_2_ availability, that is, be relatively more ‘O_2_ diffusion limited’, compared to recreational swimmers. We also hypothesized muscle oxidative capacity and relative resistance to O_2_ diffusion would be associated with markers of training volume and swimming performance.

## 2 METHODS

### 2.1 Ethical approval

All participants were informed about the study's aim and procedures, including associated risks and benefits. Written informed consent was obtained before participants started the study. All procedures conformed to the standards set by the *Declaration of Helsinki*, except for registration in a database, and were approved by the local Ethics Committee (Besta 64‐19/07/2019).

### 2.2 Participants

Twenty‐eight swimmers were enrolled and allocated to one of five different categories according to a classification framework based on individual exercise backgrounds and athletic abilities (Mckay et al., 2022): Tier 1 (‘recreational’), Tier 2 (‘trained’), Tier 3 (‘national level’), Tier 4 (‘international level’) and Tier 5 (‘world class’). Participants were recruited in different countries, between June 2023 and September 2024. Swimmers were included if they trained at least 4 km week^−1^ and front‐crawl was their preferred style. All swimmers were familiar with competing over 100 m.

### 2.3 Experimental procedures

Participants were evaluated on two separate, non‐consecutive days. They were instructed to abstain from strenuous exercise before the test and avoid consuming caffeine for at least 24 h before testing. During the first session, swimmers performed a 100 m time trial in a 50 m swimming pool. On the second visit, participants were initially familiarized with the NIRS protocol before undergoing the assessment.

### 2.4 FINA points

FINA points were obtained from the public access database ‘swimstats.net/finacalculator’. This database contains information about registered races and is in accordance with the FINA rules. The calculation of FINA points is based on the formula: 1000 × (World Record time (s)/swim time (s)^3^) (FINA, Word Aquatics). In this study, the FINA point values for 2023 were considered for the analysis.

### 2.5 Swimming performance

Swimming performance was assessed via a 100 m freestyle trial in a 50 m pool. For Tiers 3, 4 and 5, 100 m times were sourced from official competition results. For Tier 2, following a moderate‐intensity standardised warm‐up, 100 m times were recorded manually with a stopwatch during a simulated race. Athletes were encouraged to exert maximum effort throughout the trial to help elicit their best performance.

### 2.6 Training volume

Training volume was quantified as the total weekly swimming distance, expressed in kilometres. The total weekly swimming distance was self‐reported by the coach of each athlete, who tracked the distance during each training session. The weekly training volume was calculated by summing the swimming distances across all sessions within a given week. Tier 4/5 swimmers performed around 2 h week^−1^ of strength training, including single‐ and multi‐joint exercises for both upper and lower body. Tier 1/2 did not perform any specific strength training exercise. This information for Tier 3 swimmers was not available.

### 2.7 Muscle O_2_ uptake recovery rate constant by NIRS

The $m{\dot{V}}_{O_{2}}$ recovery rate constant (*k*) of m. triceps brachii was measured with participants laying prone on an examination bed. A wireless portable continuous‐wave NIRS device (Train.Red Plus, Train.Red B.V., Einsteinweg, the Netherlands) was placed on the participant's skin over the m. triceps brachii, with positioning based on Surface EMG for Non‐Invasive Assessment of Muscles (SENIAM)’s recommendations. This NIRS device is a small (1.2 × 4.4 × 5.9 cm) and lightweight (24 g) sensor with light emitting diodes (760, 850 nm) and a line receiver consisting of 64 pixels, at a mid‐range pixel distance of 35 mm (da Mota Moreira et al., 2023). Changes in the tissue saturation index (TSI) were sampled at 10 Hz using spatially resolved spectroscopy (Adami et al., 2017). All participants had an adipose tissue thickness (ATT) lower than 1 cm, which helped ensure that muscle chromophores contributed to the absorption of the near‐infrared light (Barstow, 2019). ATT was determined at the site of NIRS probes by skinfold calliper (Holtain Ltd, Crymych, UK). A 13 × 85 cm rapid‐inflation pressure cuff (SC12D; Hokanson, Bellevue, WA, USA) was placed proximally on the same upper limb and attached to an electronically controlled rapid cuff‐inflator (E20; Hokanson). Participants rested in a prone position with arms by their sides for 2–3 min to ensure stable baseline values. Subsequently, a prolonged arterial occlusion was induced (∼300 mmHg) until TSI reached a plateau (typically ∼3 min), after which the cuff was immediately deflated, and muscle reoxygenation was recorded until resting values occurred (typically 5 min). The physiological range of TSI values (i.e. physiological normalization, PN) was defined as between the deflection point (termed TSI min) and the maximum value reached during the reperfusion phase (termed TSI max) (Pilotto et al., 2022). Then, participants were asked to perform cyclical flexion‐extension of their elbow against a fixed resistance to reduce TSI down to ∼50% PN. Immediately after, a series of brief arterial occlusions were performed by rapidly inflating and deflating the pressure cuff. The rate of decline in TSI (% s^−1^) during each intermittent occlusion was measured and plotted against time to fit an exponential recovery rate constant (*k*, min^−1^) of muscle O_2_ consumption (Motobe et al., 2004). The investigator modulated the duration and timing of the repeated occlusions and releases to maintain TSI in two different ranges: 10–20% of PN (LOW) and 50–60% of PN (HIGH). The HIGH range was chosen to ensure that the intermittent occlusion was performed in the condition of well oxygenated muscle, and the LOW range was selected to detect $m{\dot{V}}_{O_{2}}$ recovery *k* in poorly oxygenated conditions (Pilotto et al., 2022). Subsequently, *k* values were determined within each condition (*k*_HIGH_ and *k*_LOW_) and the difference between conditions provided ∆*k*. All *k* values are reported as the mean of two repeated measurements.

### 2.8 Statistical analyses

Descriptive data are presented as a mean ± SD. Because there was only one Tier 5 participant, Tier 4 and Tier 5 were combined (Tier 4/5). The same approach was used for swimmers in Tier 1 and Tier 2 (Tier 1/2). Normal distribution was verified with the Shapiro–Wilk test. A paired Student's *t*‐test was used to test within individual differences between *k* values obtained from repeated trials; coefficient of variation (CV) and intraclass correlation coefficient (ICC) were used to assess within‐subject test–retest reliability in *k* values. Additionally, the 95% confidence of interval (CI) for the ICC was also calculated. One‐way analysis of variance (ANOVA) was used to assess differences among groups (Tier 4/5, Tier 3, Tier 1/2). Tukey's test was used for *post hoc* analysis when a significant main group effect was observed. Pearson's product‐moment correlation coefficient (*r*) was used to examine associations between variables. Results were considered significant at *P *< 0.05. All statistical analyses were performed using the software package Prism 10.0 (GraphPad, Software, Boston, MA, USA).

## 3 RESULTS

### 3.1 Participants

Table 1 shows participant characteristics. There were three Tier 1, five Tier 2, twelve Tier 3, seven Tier 4 and one Tier 5 participants. The weekly training volume is also shown in Table 1. Training volume increased with Tier group.

**TABLE 1:** Participants characteristics and training information.

Groups	Age (years)	Body mass (kg)	Height (m)	BMI (kg m^−2^)	Volume of training (h week^−1^)
Tier 4/5 (*n* = 8)	24.3 ± 4.4	77.3 ± 11.9	1.83 ± 0.1	22.9 ± 1.6	34.0 ± 5.5
Tier 3 (*n* = 12)	23.8 ± 6.1	74.1 ± 9.4	1.78 ± 0.1	23.0 ± 1.8	35.5 ± 11.6a
Tier 1/2 (*n* = 8)	27.8 ± 1.9	67.1 ± 9.9	1.73 ± 0.0	22.4 ± 2.3	6.4 ± 1.8a,b

Data are presented as means ± SD. ^a^Significantly different from Tier 4/5. ^b^Significantly different from Tier 3. BMI, body mass index.

### 3.2 FINA points and 100 m performance

**FIGURE 1:**
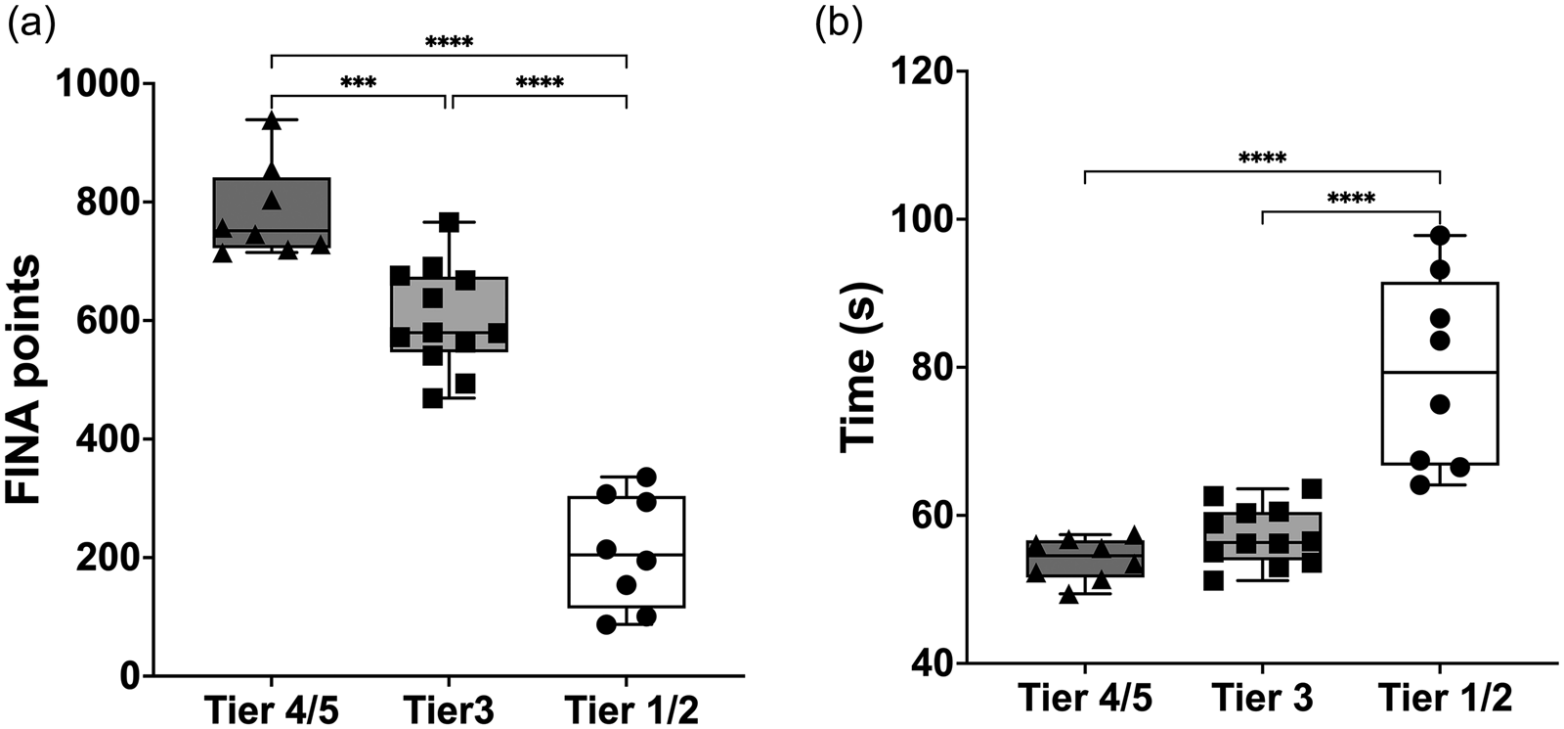
Comparative analysis of FINA points and performance times in swimmer populations. (a) The distributions of FINA points (International Swimming Federation points) across swimmer populations investigated. (b) Performance time for completing the 100 m freestyle stroke among swimmer populations. Data are presented as means ± SD. Tier 1/2, white box, black circles; *n* = 8; Tier 3, light grey, black squares; *n* = 12; Tier 4/5, dark grey, black triangles; *n* = 8. ***P < 0.05, **** *P *< 0.0001, significantly different among groups.

FINA points are shown in Figure 1a. One‐way ANOVA found differences in FINA points among the groups (*F*(2,25) = 91.89; *P *< 0.0001). *Post hoc* analysis revealed significant differences between Tier 4/5 and Tier 3 (*P* = 0.0004), Tier 3 and Tier 1/2 (*P *< 0.0001), and Tier 4/5 and Tier 1/2 (*P *< 0.0001).

Performance time for the 100 m trial is shown in Figure 1b. One‐way ANOVA found differences in completion time among groups (*F*(2,25) = 28.34; *P *< 0.0001). *Post hoc* analysis indicated significant differences between Tier 4/5 and Tier 1/2 (*P *< 0.0001), with Tier 4/5 completing the 100 m ∼35% faster than Tier 1/2. Additionally, Tier 3 was ∼31% faster than Tier 1/2 (*P *< 0.0001).

### 3.3 Triceps brachii muscle oxidative and O_2_ diffusive function

*k*_HIGH_ was 2.70 ± 0.64 and 2.63 ± 0.52 min^−1^ for the first and second repetitions, respectively. No significant differences were found between the two repeated measurements (*P* = 0.6951). Similarly, *k*_LOW_ was 1.72 ± 0.47 and 1.85 ± 0.44 min^−1^ for the first and second trials, respectively, with no significant differences (*P* = 0.3840). The CV and ICC for *k*_HIGH_ (ICC = 0.84, 95% CI: 0.71–0.95; CV = 11%) and *k*_LOW_ (ICC = 0.86; 95% CI: 0.61–0.94; CV = 15%) demonstrated good reliability.

**FIGURE 2:**
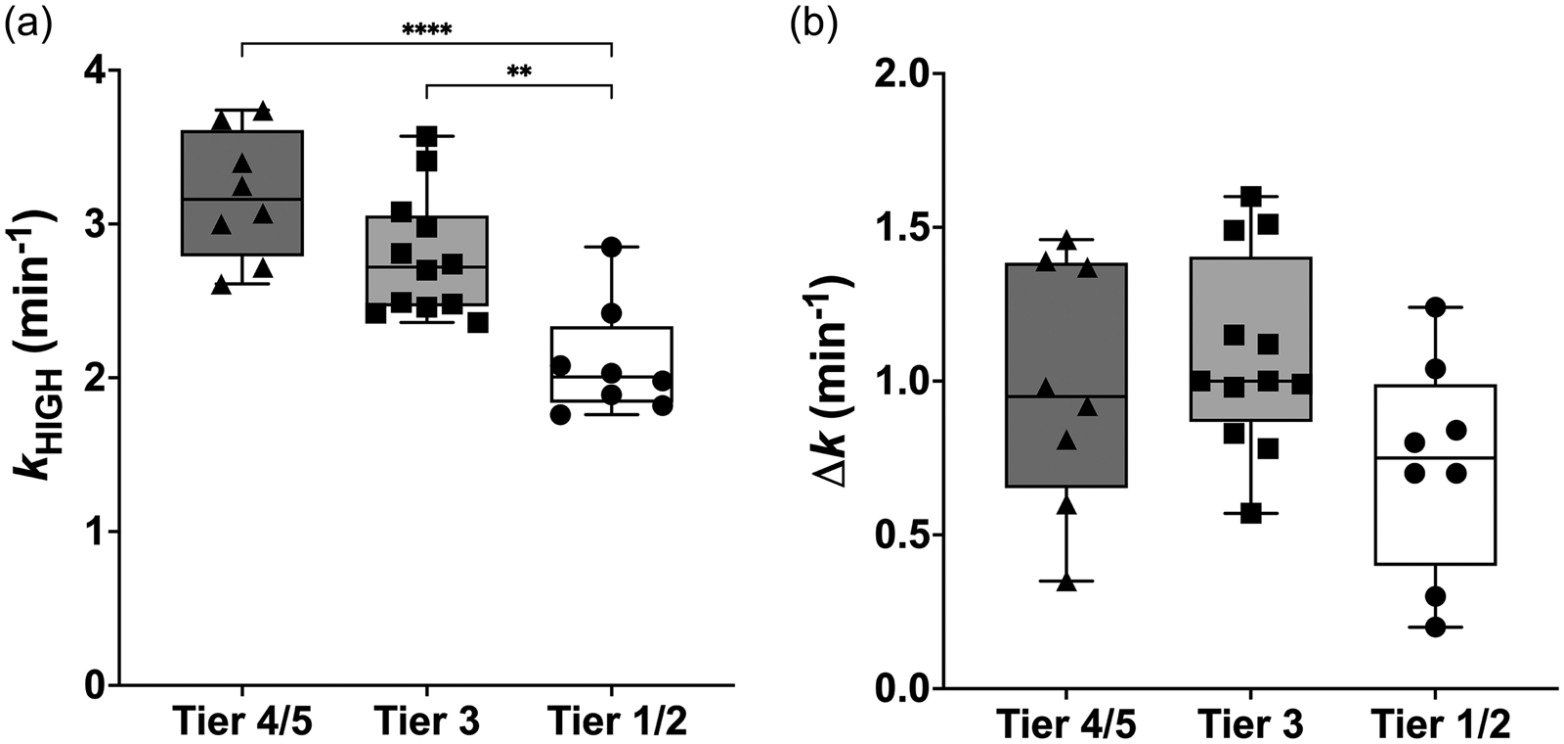
Comparative analysis of muscle oxidative and O_2_ diffusive function across swimmer populations. (a) Muscle oxygen consumption ($m{\dot{V}}_{O_{2}}$) recovery rate constant (*k*) by NIRS under conditions of high O_2_ availability (*k*_HIGH_). (b) Differential recovery rate constant (Δ*k*), calculated as the difference between *k*_HIGH_ and *k*_LOW_ across swimmer populations (where *k*_LOW_ is the $m{\dot{V}}_{O_{2}}$ recovery rate constant obtained under conditions of limited O_2_ availability). Data are presented as means ± SD. Tier 1/2, white box, black circles; *n* = 8; Tier 3, light grey, black squares; *n* = 12; Tier 4/5, dark grey, black triangles; *n* = 8. ***P < 0.05, ****P < 0.0001, significantly different among groups.

*k*_HIGH_ was significantly greater (*P *< 0.0001) than *k*_LOW_ in all athletes. One‐way ANOVA revealed significant differences in *k*_HIGH_ among Tier groups (*F*(2,25) = 15.59; *P *< 0.0001). *Post hoc* analysis indicated that *k*_HIGH_ was significantly greater in Tier 4/5 and Tier 3 compared to Tier 1/2 (*P *< 0.0001 and *P* = 0.0023, respectively) (Figure 2a). A main effect of group was observed for *k*_LOW_ (*F*(2,25) = 5.07; *P* = 0.0142). *Post hoc* analysis indicated that *k*_LOW_ was significantly greater in Tier 4/5 compared to Tier 1/2 (*P* = 0.0123). ∆*k* was not different among groups (*F*(2,25) = 2.568; *P* = 0.097) (Figure 2b).

### 3.4 Correlations

**FIGURE 3:**
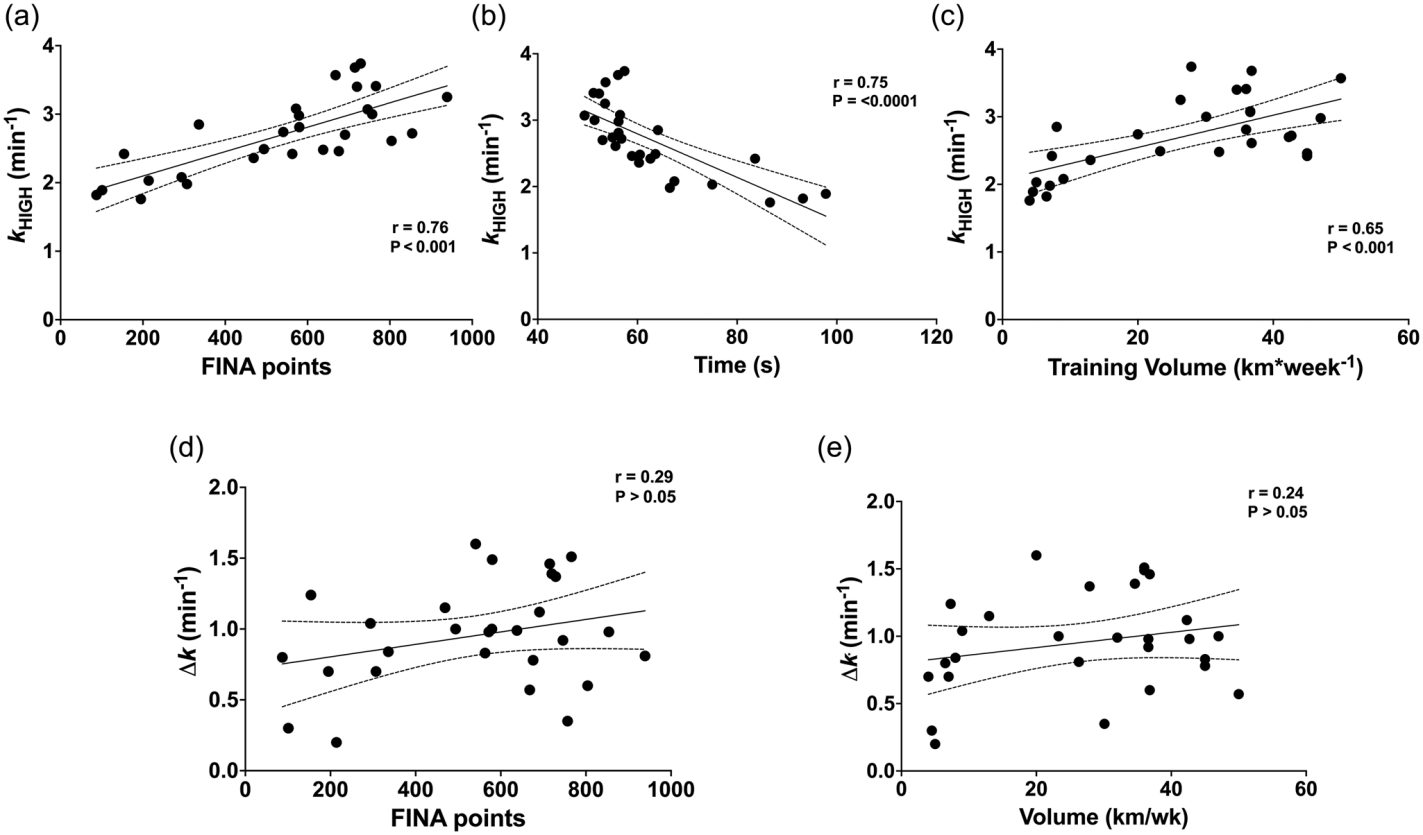
(a–c) Correlation between muscle oxidative capacity (*k*_HIGH_) and FINA points (a), time to complete a 100 m trial (b), and total weekly training volume (c). (d) Correlation between the differential recovery rate constant (Δ*k*) and FINA points. (e) Correlation between between Δ*k* and total weekly training volume. The paired dashed line represents the 95% confidence interval. Points (black circle) represent individual data. For each plot, linear regression equations, Pearson's coefficient (*r*), and *P*‐values are reported. *n* = 28.

Figure 3 shows significant correlations between *k*_HIGH_ and FINA points (*r* = 0.76; *P *< 0.0001), *k*_HIGH_ and 100 m performance (*r* = 0.75; *P *< 0.0001), and *k*_HIGH_ and training volume (*r* = 0.65; *P* = 0.0002). In contrast, no significant correlation was observed between ∆*k* and FINA points (*P* = 0.14) or training volume (*P* = 0.23).

## 4 DISCUSSION

This study aimed to evaluate oxidative and O_2_ diffusive function in the m. triceps brachii of swimmers ranging in ability from recreational to world class, and their associations with swimming performance. The main finding was that international and world class swimmers had a significantly greater m. triceps brachii oxidative capacity (*k*_HIGH_) than recreational athletes. Additionally, we observed a positive correlation between non‐invasive estimates of muscle oxidative capacity and training volume or measures of swimming performance. In contrast, our non‐invasive estimate of relative resistance to O_2_ diffusion (Δ*k*) was not different among swimmers of different abilities. This suggests that the high oxidative capacity in m. triceps brachii of international and world class swimmers was supported by a proportionally similar adaptation in the capillary network and convective O_2_ delivery system, sufficient to prevent a greater limitation to muscle oxidative function caused by diffusive O_2_ flow compared to swimmers in lower tiers. Thus, we did not find a relationship between Δ*k* and training volume or performance in recreational to world class swimmers.

In healthy trained and untrained humans, oxidative capacity of the primary muscles of locomotion is strongly correlated with whole‐body ${\dot{V}}_{O_{2}\max}$ and endurance exercise performance (Hood et al., 2011; Hoppeler et al., 1985). Moreover, data from Buso et al. (2019) emphasize that a sedentary lifestyle is associated with diminished muscle oxidative capacity. Muscle oxidative capacity is commonly assessed using *ex vivo* techniques, including analysing muscle biopsy samples for enzyme activity or mitochondrial O_2_ flux through isolated mitochondrial preparations or permeabilized muscle fibres (Brand & Nicholas, 2011; Perry et al., 2013). However, non‐invasive in vivo approaches, including NIRS, have emerged as a valuable tool capable of assessing $m{\dot{V}}_{O_{2}}$ recovery rate constant, *k*, which is strongly associated with muscle oxidative capacity (Adami & Rossiter, 2018; Adami et al., 2017; Grassi & Quaresima, 2016; Ryan et al., 2014, 2013). In our study, *k*_HIGH_ in the m. triceps brachii was lower in Tier 1/2 compared with Tiers 3 and 4 populations and one Tier 5 swimmer, consistent with the expectation that oxidative capacity is greater in primary locomotor muscles of high performance compared with recreational athletes. It is of interest that we found *k*_HIGH_ values of m. triceps brachii of Tier 4/5 swimmers in the range of ∼3–4 min^−1^, which is similar to that found in vastus lateralis of trained runners or cyclists Brinzedine et al. (2013); this is despite the fact that upper limb muscles typically have a lower type I fibre proportion and lower oxidative capacity than vastus lateralis. This suggests that high oxidative capacity in the m. triceps brachii of swimmers may represent a selective advantage and/or training adaptation that benefits swimming performance. By comparison, Brinzedine et al. (2013) showed that endurance athletes (runners/cyclists) have greater *k* in the vastus lateralis (the primary locomotor muscles for their sports) than inactive controls. Adami and Rossiter (2018) summarized available evidence of *k* in a range of muscles across the spectrum of human performance – from spinal cord injury and chronic disease to endurance trained athletes, confirming a remarkable ∼6‐fold difference in *k* among the populations and muscles examined. We found an ∼1.5‐fold difference in m. triceps brachii *k*_HIGH_ between Tier 1/2 and Tier 4/5 swimmers in our study, which is consistent with the magnitude of endurance training‐induced differences observed in other muscle groups, for example, ∼1.7‐fold (Adami & Rossiter (2018).

As expected, Tier 4/5 swimmers had a greater training volume (∼35 km/week) than Tier 1/2 (∼7 km/week). In contrast, Tier 4/5 swimmers demonstrated no significant differences in training volume compared with Tier 3. This aligns with previous studies suggesting that increased training volume did not consistently enhance swimming performance when a high‐performance level is reached (González‐Ravé et al., 2021; Seiler, 2010). These data support our hypothesis that differences in *k*_HIGH_ among tiers might be attributable to differences in training volume. However, naturally, we cannot rule out self‐selection and genetic predisposition in our study, which contains a relatively limited sample size. Nevertheless, it is evident that endurance training volume plays a key role in enhancing mitochondrial volume/density and oxidative function, which might underlie the observed differences in *k*_HIGH_ among groups in our study. Our data are consistent with a large body of historical data showing a relationship between endurance exercise training and changes in skeletal muscle oxidative function (Baldwin et al., 1972). For example, Granada et al. (2016) showed an association between training volume and mitochondrial protein content or oxidative capacity in human skeletal muscle. In a recent systematic review, Mølmen et al. (2024) found that both greater training frequency and greater training volume resulted in a pronounced increase in mitochondrial volume/density. It should be acknowledged that the intensity of the exercise stimulus can modulate training‐induced increases in mitochondrial volume/density (MacInnis et al., 2019). Unfortunately, we were not able to collect more details about training, other than total volume. Further study is required to understand the role of intensity in relation to muscle oxidative and O_2_ diffusive function. As previously mentioned, Tiers 3 and 4/5 swimmers usually include several strength training sessions in their weekly routine. Thus, we can speculate that this strength/power training did not prevent an increase in muscle oxidative capacity (Cardinale et al., 2017).

It should be noted that in our study we did not measure physiological parameters related to convective O_2_ delivery to the muscle, a main determinant of maximal muscle O_2_ consumption. However, muscle mitochondrial density also facilitates O_2_ extraction (Skattebo et al., 2020), particularly during small muscle mass exercises, such as upper‐body exercises (Cardinale et al., 2019). Thus, we infer that the greater swimming performance in Tier 4/5 and Tier 3 was due in part to greater muscle oxidative metabolism.

Here we evaluated swimming performance using a 100 m trial. As expected, Tier 4/5 had faster 100 m trial times than Tier 1/2. While muscle oxidative capacity is thought to be a key variable mediating endurance exercise performance, it is, of course, not the only contributing factor. Swimming performance is a complex outcome influenced by multiple factors, including physiological, biomechanical and anthropometric characteristics (Lätt et al., 2010; Morais et al., 2021). Literature reports that elite swimmers have superior physiological characteristics that contribute to determining a faster performance time. Data suggest that the ability to recruit a higher proportion of fast‐twitch motor units may underlie the performance characteristics of elite athletes, especially in sprint swimmers (Bellinger et al., 2022). A greater muscle oxidative capacity may confer fatigue resistance in these motor units (McDougall et al., 2023), allowing increased engagement of higher‐order motor units into the exercise performance. Biomechanical efficiency also plays a crucial role in swimming performance. Elite swimmers are often better able to maintain optimal stroke mechanics during performance trials, which reduces drag and facilitates propulsion in the water. This efficiency is supported by anthropometric characteristics, such as longer limb lengths and body composition, contributing to better hydrodynamics (Morais et al., 2013). Nevertheless, high muscle oxidative capacity may also contribute to the maintenance of biomechanical efficiency, again through enhanced fatigue resistance. Collectively, several factors are needed to explain the difference in performance among groups in our study, but m. triceps brachii oxidative capacity appears strongly associated with swimming performance from recreational to world class athletes.

Portable and wearable devices suitable for field‐based measurements have revolutionized the study of exercise physiology by providing real‐time, non‐invasive and spatially localized measurements of physiological determinants of performance (Perrey et al., 2024). Among the others, NIRS has been increasingly used in sport sciences to assess various physiological parameters related to exercise performance (Barstow, 2019; Grassi & Quaresima, 2016; Hamaoka & McCully, 2019; Perrey et al., 2024). A recent review reported more than 190 studies related to 37 different sporting activities used NIRS to investigate muscle oxygenation changes (Perrey et al., 2024). Nevertheless, only four studies investigated muscle oxygenation changes or muscle adaptations to training in swimmers by NIRS, and none investigated the muscle oxidative and O_2_ diffusing capacity. This highlights the novelty of our study, posing a basis for future studies to test the potential benefits to athletes and coaches to use NIRS to monitor muscle oxidative and diffusive adaptations during the competition.

**FIGURE 4:**
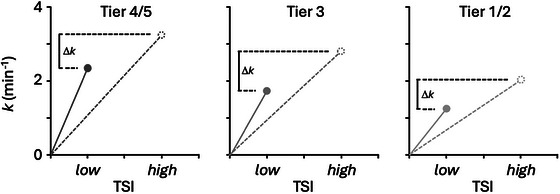
Theoretical representation of muscle O_2_ diffusion by NIRS in swimmers ranging in ability from international/world class (Tier 4/5; a), national (Tier 3; b) recreational/trained (Tier 1/2; c). The slope of each line connecting the measured *k* value to the origin represents the absolute rate of O_2_ diffusion. In every group, the absolute value of *k*_LOW_ is lower than *k*_HIGH_, implying that the limits to muscle O_2_ diffusion have been revealed in *k*_LOW_ by the experimental manipulation of TSI – otherwise *k*_LOW_ and *k*_HIGH_ would be equal. Because *k*_HIGH_ is greater in Tier 4/5 swimmers than in lower tiers, and because Δ*k* is similar among groups, this implies that the absolute muscle O_2_ diffusive capacity (i.e. the theoretical slope of *k*_LOW_ to the origin) is greater in Tier 4/5 swimmers. As such, the finding that Δ*k* is similar among groups implies a similar degree of adaptation in both muscle oxidative and O_2_ diffusing capacities in high tier swimmers, such that relative resistance to O_2_ diffusion to support muscle oxidative capacity is similar among groups.

Another novel aspect of this study was to assess relative resistance to O_2_ diffusion in the same muscles and participants as oxidative capacity. This was achieved using the approach developed by Pilotto et al. (2022), where $m{\dot{V}}_{O_{2}}$ recovery *k* was assessed under O_2_‐limited (*k*_LOW_) and non‐limited (*k*_HIGH_) conditions. This is a NIRS‐based modification of an approach pioneered by Wagner et al. (e.g. Roca et al., 1992) to quantify convective and diffusive O_2_ flow at ${\dot{V}}_{O_{2}\max}$ and thereby apportion weightings to the variable potentially limiting oxidative function and endurance exercise performance. By restricting capillary O_2_ availability in *k*_LOW_, this technique can establish the relative sensitivity of muscle oxidative capacity to O_2_ diffusion. A large difference between *k*_HIGH_ and *k*_LOW_ (a large Δ*k*) is associated with reduced muscle capillary density (Pilotto et al., 2022), and implies a relatively greater resistance, or limitation, to oxidative function by O_2_ diffusion. These effects are stylized in Figure 4, using the group mean values obtained in this study. In Figure 4, the slope of each line connecting the measured *k* value to the origin represents the rate of O_2_ diffusion for a given muscle oxidative capacity. Since, in every case, the absolute value of *k*_LOW_ is lower than *k*_HIGH_, we can infer that the limits to muscle O_2_ diffusion were revealed in *k*_LOW_ by the experimental manipulation of TSI – otherwise O_2_ diffusive flow would increase to the degree that would result in *k*_LOW_ and *k*_HIGH_ being equal. As the theoretical Figure 4 reveals, the steepest slope, representing muscle O_2_ diffusive capacity (i.e. the slope of *k*_LOW_ to the origin), is greater with increasing swimming performance, but Δ*k* remains similar among groups. A similar Δ*k* among groups implies that the relative resistance to O_2_ diffusion is similar.

Although this NIRS‐based technique is not able to provide absolute rates of muscle O_2_ diffusion, it does allow us to interpret the relative resistance to O_2_ diffusion through interpretation of Δ*k*. NIRS is not able to measure absolute values capillary $P_{O_{2}}$ (as would be required for the *x*‐axis of Figure 4) or absolute rates of ${\dot{V}}_{O_{2}}$ (as would be required for the *y*‐axis of Figure 4) to calculate absolute rates of O_2_ diffusion using the Wagner approach (i.e. the absolute slopes of the lines in Figure 4). Therefore, as we have previously argued, it is not valid to use the NIRS approach to construct a quantitative and statistical comparison of the slopes represented in Figure 4 (Porcelli et al., 2023). Instead Figure 4 represents a theoretical construct to help explain the interpretation of the Δ*k* variable, which represents the relative resistance to O_2_ diffusion. This interpretation is also supported by our previous work (Pilotto et al., 2022), demonstrating that Δ*k* is negatively associated with anatomic variables that support diffusion (such as capillary density). Thus, because Tier 4/5 swimmers have greater muscle oxidative capacity but similar Δ*k* to swimmers in lower tiers, it implies that the Tier 4/5 swimmers also have greater absolute O_2_ diffusive capacity than lower tier swimmers. Adaptations that may support this increased rate of O_2_ diffusion in Tier 4/5 swimmers include increased capillary density, increased mean transit time, greater diffusional surface area, reduced diffusion distances, and better maintained capillary $P_{O_{2}}$ at ${\dot{V}}_{O_{2}\max}$.

Importantly, the demands for diffusive O_2_ flow have the potential to be greater in muscles with increased oxidative capacity. This is because endurance exercise training pushes the potential stenosis in oxidative energy provision from inside the muscle (e.g. oxidative enzyme activity) to outside the muscle (e.g. factors such as capillary density or fraction of capillary‐to‐red‐cell apposition as well as potentially myoglobin concentration). As such, we anticipated that higher Tier swimmers would have a greater Δ*k*, as training‐induced intramuscular adaptations supporting oxidative capacity were expected to outstrip other muscular adaptations supporting O_2_ diffusion. In contrast to our hypothesis, our data showed no effect of tier on Δ*k*, suggesting relative resistance to diffusive O_2_ flow in m. triceps brachii was similar among recreational, national, international and world class swimmers. However, the study may be underpowered to identify these differences. In a secondary analysis, by considering Tier 3–5 as one group, we found a lower Δ*k* in Tier 1/2 swimmers (1.05 ± 0.34 vs. 0.73 ± 0.34 min^−1^; *P* = 0.033 by 2‐tailed *t*‐test). Interpreting this, with reference to Figure 4, we see that Δ*k* is lowest in Tier 1/2. This group may be less sensitive to diffusion limitation – not because of greater physiological adaptations that support diffusion, such as capillary density, but because these swimmers have a lower absolute muscle oxidative capacity (*k*_HIGH_). This finding in secondary analysis is consistent with the notion that skeletal muscles with a lower oxidative capacity are less sensitive to O_2_ diffusion limitation. The implication of this finding is that training designed to enhance adaptations of O_2_ diffusion in higher tier swimmers could lead to significant gains in endurance swimming performance. Nevertheless, we were unable to show a significant relationship between Δ*k* and measures of swimming performance and further study is needed to confirm or refute these findings.

### 4.1 Limitations

In this study we included both men and women, and it is known that sex affects the integrative response to exercise (Ansdell et al., 2020). Thus, we cannot exclude that some differences among tiers might be explained by including both men and women. Unfortunately, we lack the statistical power to investigate sex differences and future research is needed to address this issue.

Oxidative and O_2_ diffusive function in the m. triceps brachii of recreational to world class swimmers were determined in vivo by NIRS. This approach is an indirect assessment of the primary variables. While oxidative function has been previously evaluated by NIRS in different muscle groups or subjects (Adami & Rossiter, 2018), non‐invasive estimation of diffusion limitations to O_2_ flux has been validated on m. vastus lateralis in healthy moderately trained subjects alone. However, the physiological assumptions of the NIRS approach where changes in *k* values estimate relative O_2_ diffusion should be valid independent of the muscle group or the participant characteristics. Future studies are needed to further investigate these methods.

### 4.2 Conclusion

This was the first study to use NIRS to assess in vivo muscle oxidative capacity and relative resistance to muscle O_2_ diffusion in m. triceps brachii. We found this technique to have acceptable test–retest reliability, suggesting that NIRS could form a useful in vivo assessment of oxidative function in this muscle group. We found that oxidative capacity varied significantly among swimmers ranging in ability from recreational to world class, and that oxidative capacity, as expected, was positively associated with both training volume and swimming performance. Contrary to our hypothesis, however, we were unable to detect differences in relative resistance to intramuscular O_2_ diffusion in higher tier swimmers, suggesting that endurance training adaptations to support oxidative capacity were matched well to adaptations to support O_2_ diffusion in a major locomotor muscle for swimming.

## AUTHOR CONTRIBUTIONS

Simone Villanova and Simone Porcelli conceived and designed research; Simone Villanova, Elisa Pastorio, Alessio Marciano and Daniele A. Cardinale performed experiments; Simone Villanova performed statistical analyses; Simone Villanova, Elisa Pastorio, Alessio Marciano, Harry B. Rossiter, Daniele A. Cardinale and Simone Porcelli interpreted results of experiments; Simone Villanova and Simone Porcelli drafted the manuscript; Simone Villanova, Elisa Pastorio, Andrea M. Pilotto, Alessio Marciano, Valentina Quaresima, Alessandra Adami, Harry B. Rossiter, Daniele A. Cardinale and Simone Porcelli edited and revised the manuscript. All authors have read and approved the final version of this manuscript and agree to be accountable for all aspects of the work in ensuring that questions related to the accuracy or integrity of any part of the work are appropriately investigated and resolved. All persons designated as authors qualify for authorship, and all those who qualify for authorship are listed.

## CONFLICT OF INTEREST

The authors declare that the research was conducted without commercial or financial relationships that could create a conflict of interest. H.R. reports consulting fees from the NIH RECOVER‐ENERGIZE working group (1OT2HL156812), and is involved in contracted clinical research with Astellas, GlaxoSmithKline, Genentech, Intervene Immune, Mezzion, Novartis, Regeneron, Respira, and United Therapeutics. He is a visiting Professor at the University of Leeds, UK.

## Data Availability

All data generated and analysed during this study are available from the corresponding author upon reasonable request. The data are not publicly available owing to privacy or ethical restrictions.
